# Answering the Call to Accelerate the Elimination of Cervical Cancer

**DOI:** 10.1055/s-0039-1679864

**Published:** 2019-02

**Authors:** Joanna M. Cain

**Affiliations:** 1 School of Medicine, Department of Obstetrics and Gynecology, University of Massachusetts, Massachusetts, United States

The opportunity to improve the lives and survival rates of women through the elimination of cervical cancer is a dream that every obstetrician-gynecologist, every women's health specialist, and our societies must embrace. I can never forget the anguished faces of the 4-year-old son and of the husband of one of my first young patients dying of cervical cancer because it had been just “too far and too much money” to go for cervical screening. We now have the means to prevent that tragedy for other families.

Cervical cancer causes more deaths than maternal mortality in many regions of the globe, and kills well over a quarter million women per year, most in low- and middle-income countries or in rural and low-income areas within countries.^1^ Most of these deaths could be prevented with access to human papillomavirus (HPV) immunization and cervical screening services. Without scaling up our action, the number of deaths due to this condition will grow in the future.

When Dr. Tedros, the Director-General of the World Health Organization (WHO), gave the call to action at the 2018 World Health Assembly, it set off a series of actions. There was a major focus on HPV immunization, on cervical screening and treatment, and on cancer care at the 2018 Rio International Federation of Gynecology and Obstetrics (FIGO) World Congress. At that meeting, the FIGO joined the initiative with a “Global Declaration on Cervical Cancer Elimination”, uniting the FIGO member societies with the WHO, the United Nations Population Fund (UNFPA), and with other international partners in coordinating actions locally and regionally to assure that “all women and girls, in particular those in low-income countries, have equal access to life-saving prevention technologies and services.”^2^

As part of that Declaration, FIGO and its 132 member societies committed to promote certain actions that require societies and their members to step forward within their local and regional settings. These include support for rolling out HPV vaccines, advocacy for national cervical cancer elimination and treatment strategies, and support for capacity-building efforts to expand knowledge and skills. Whether it is working one-on-one with patients, volunteering, educating the community and policy makers, or by means of other initiatives, these are actions that we can take now in our practices, as well as in our local areas and societies, and share the information with collaborations and partnerships regionally and globally.

For global support, the WHO has convened seven working groups including two on prevention and treatment to outline a roadmap for the global initiative. The FIGO has two representatives on these small roadmap groups. The first meeting occurred in December 2018 to define areas such as what targets should be set globally (what is the percentage of women screened at least once in a lifetime, and by what date, for example), how to make HPV vaccines feasible in resource-poor areas, which WHO recommendations need updating, which laboratory standards support might be possible and needed, and other areas required to provide the global infrastructure to support countries and societies as they tackle this problem. The various roadmaps will be coordinated over 2019 with the goal of creating a plan and a budget for the WHO to put forward in the fall of 2019. While this effort is important, it is still dependent on the work on national and regional societies and advocates to make this dream a reality, and those efforts should move forward now.

Having the chance to actually prevent a cancer and effectively treat those missing immunization in order to eliminate such a major cause of suffering, of family disruption, and of death of women is one that we are unlikely to see again at this scale in our lifetimes. Let us hold on to this dream and make it real. There is no time to wait. Engage now, at every level, in the initiative to eliminate cervical cancer.
